# Using HPLC–DAD and GC–MS Analysis Isolation and Identification of Anticandida Compounds from Gui Zhen Cao Herbs (Genus *Bidens*): An Important Chinese Medicinal Formulation

**DOI:** 10.3390/molecules26195820

**Published:** 2021-09-25

**Authors:** Kulsoom Zahara, Yamin Bibi, Saadia Masood, Sobia Nisa, Abdul Qayyum, Muhammad Ishaque, Khurram Shahzad, Waseem Ahmed, Zahid Hussain Shah, Hameed Alsamadany, Seung-Hwan Yang, Gyuhwa Chung

**Affiliations:** 1 Department of Botany, PMAS-Arid Agriculture University Rawalpindi, Rawalpindi 46300, Pakistan; kulsoomzahara@gmail.com (K.Z.); dryaminbibi@uaar.edu.pk (Y.B.); mishaque270@gmail.com (M.I.); 2 Department of Statistics & Mathematics, PMAS-Arid Agriculture University Rawalpindi, Rawalpindi 46300, Pakistan; saadia.masood@uaar.edu.pk; 3 Department of Microbiology, The University of Haripur, Haripur 22620, Pakistan; sobia@uoh.edu.pk; 4 Department of Agronomy, The University of Haripur, Haripur 22620, Pakistan; 5 Department of Plant Breeding and Genetics, The University of Haripur, Haripur 22620, Pakistan; kshahzad@uoh.edu.pk; 6 Department of Horticulture, The University of Haripur, Haripur 22620, Pakistan; dr.waseemahmed@uoh.edu.pk; 7 Department of Plant Breeding and Genetics, PMAS Arid Agriculture University, Rawalpindi 46300, Pakistan; shahzahid578@hotmail.com; 8 Department of Biological Sciences, King Abdul Aziz University, Jeddah 21589, Saudi Arabia; halsamadani@kau.edu.sa; 9 Department of Biotechnology, Chonnam National University, Gwangju 59626, Korea; ymichigan@chonnam.ac.kr

**Keywords:** Gui Zhen Cao, traditional Chinese medicine, linoleic acid, *Bidens bipinnata*

## Abstract

Gui Zhen Cao is an herbal formulation that has been documented in Chinese traditional medicine as a remedy for diarrhea, dysentery, inflammation, and toxicity. The sources of this formulation (*Bidens pilosa* L., *Bidens biternata* (Lour.) Merr. & Sherff, *Bidens bipinnata* L.) are also listed in ethnomedicinal reports all over the world. In this study, all these plants are tested for in vitro anticandida activity. A quantitative evaluation of the phytochemicals in all these plants indicated that their vegetative parts are rich in tannins, saponins, oxalates, cyanogenic glycoside and lipids; moreover, the roots have high percentages of alkaloids, flavonoids, and phenols. The results indicated significant anticandida activity, especially for the hexane extract of *B. bipinnata* leaves which inhibited *C. albicans* (42.54%), *C. glabrata* (46.98%), *C. tropicalis* (50.89%), *C. krusei* (40.56%), and *C. orthopsilosis* (50.24%). The extract was subjected to silica gel chromatography and 220 fractions were obtained. Purification by High Performance Liquid Chromatography with Diode-Array Detection (HPLC–DAD) and Gas Chromatography tandem Mass Spectrometry (GC-MS/MS) analysis led to the identification of two anticandida compounds: dehydroabietic and linoleic acid having an inhibition of 85 and 92%, respectively.

## 1. Introduction

Since prehistoric times, human beings have used plants as a source of medicine; in fact, they were the only source of medicine until the arrival of iatrochemistry in the 16th century [1]. After that, there was a massive shift from natural to synthetic medicine; today, modern Western medicine is based on synthetic compounds. However, in several parts of world, natural medicinal systems—Ayurveda, Unani Tib and Traditional Chinese Medicine (TCM)—also exist [2].

Gui Zhen Cao is a traditional Chinese herb that is described as having detoxifying properties. The *Oriental Materia Medica* says that it clears blood stagnation and wind dampness [3]. In the book *Thousand Formulas and Thousand Herbs of Traditional Chinese Medicine*, it is reported to remove heat from the gastrointestinal tract and cure diarrhea, dysentery, and stomach ache of the heat type (Table 1). Wind-heat is a type of common cold in Chinese medical language. Sore throat, feeling warm and/or agitated (whether or not there is a fever), yellow or green-colored phlegm, and aversion to heat are some indications of wind-heat. It is also accompanied with Heat cramps and Damp Wind, both responsible for muscles fatigue.Heat cramps is a painful, brief muscle cramps that occur during excessive work in a hot environment. Whereas Damp Wind has effects similar to those of the common cold, with sore limbs, listlessness, nausea, anorexia, and diarrhea and can cause diseases like arthritis.

In the *Chinese-English Manual of Common-Used Herbs*, this formulation is said to cure the common cold, sore throat, appendicitis, centipede bite and snake bite, and prevent influenza [5]. The sources of Gui Zhen Cao are three herbs: i.e., *Bidens pilosa L.*, *Bidens biternata* (Lour.) Merr. & Sherff, *Bidens bipinnata* L. [5]. In the manual under Gui Zhen Cao, these plants are all widely reported to be used for treating different ailments all around the world (Table 2).

Various ethnobotanical reports also indicated the use of these plants for skin and vaginal infections [7,8,9,10]. Studies also reported that these plants enhanced macrophage activity against candida infection in mice [11]. The use of *Bidens* spp. for vaginitis is also documented in the literature. For example, Borges et al. [12] reported the use of its fresh juice.

Previously, the anticandida activity of *B. pilosa* was reported [11,13] but not the effects of *B. bipinnata* or *B. biternata*. Therefore, we conducted a quantitative phytochemical analysis of the reproductive and vegetative parts of Gui Zhen Cao herbs to identify the active anticandidal phytochemicals.

## 2. Results

The quantitative determination of phytochemicals in *B. pilosa*, *B. biternata* and *B. bipinnata* is presented in Table 3. This study indicated that vegetative parts (leaves, stems, and roots) are higher in tannins, oxalates, cyanogenic glycoside, and lipids; however, alkaloids, flavonoids and phenols were higher in the reproductive parts (flowers and achenes). Alkaloids were the highest in *B. biternata* (0.499 mg/100 g) compared to the other species. *B. biternata* was also found to have a higher tannin content (1090 mg/100 g) followed by *B. pilosa* (1030 mg/100 g. The amount of saponins was the highest in *B. bipinnata*. A higher phenol content was also observed in vegetative and reproductive parts of all *Bidens* species *B. bipinnata* (4.04, 5.98), *B. pilosa* (3.58, 4.98) and *B. biternata* (3.14, 4.09). 

**Figure 1:**
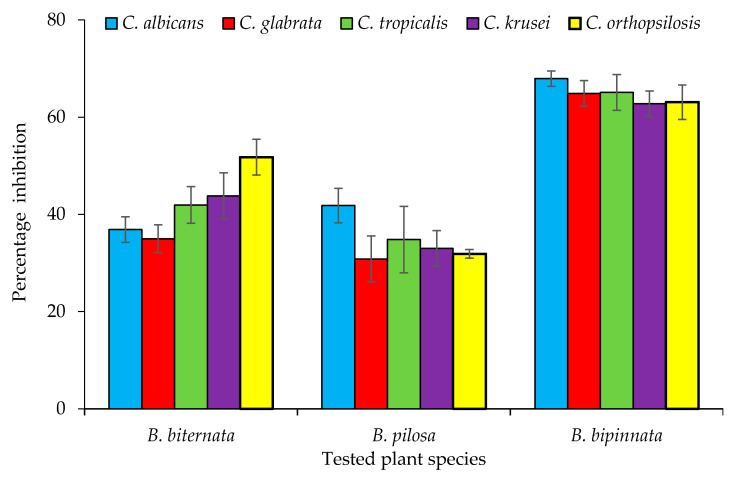
Anticandida activity of *Bidens bipinnata*, *Bidens pilosa* and *Bidens biternata*.

**Figure 2:**
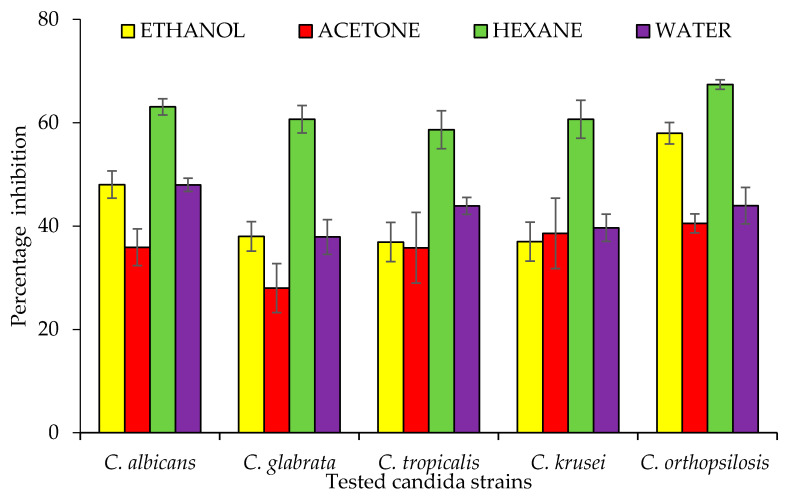
Anticandida activity of different extracts of *Bidens bipinnata*; positive control fluconazole.

**Figure 3:**
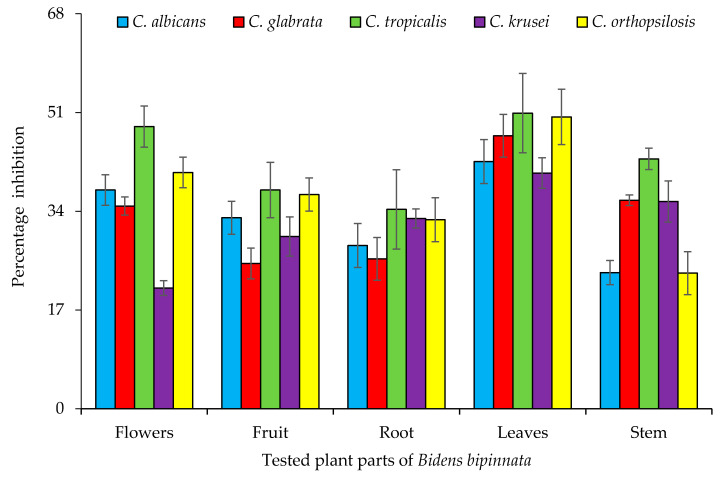
Anticandida activity of Hexane extract of different parts of *Bidens bipinnata*.

Candidiasis is a type of infection that mostly occurs in the mouth, throat, and vagina. It sometime effect organs like the kidney. In this study, three species of the genus *Bidens* (*B. pilosa*, *B. bipinnata* and *B. biternata*) were evaluated for their anticandida properties (Figure 1). The hexane extract of *B. bipinnata* appeared to be the most active with percentage inhibition of 63.09 (*C. albicans*), 60.68 (*C. glabrata*), 58.66 (*C. tropicalis*), 60.67 (*C. krusei*) and 67.40 (*C. orthopsilosis*) (Figure 2). The hexane extract of different parts of *B. bipinnata* (leaves, stem, root, flower and achene’s) were again tested for anticandida activity. The leaves’ hexane extract appeared to be the most active (Figure 3). Thus, for large scale extraction, the hexane extract of the leaves was selected. From 200 g of leaf powder in 1000 mL hexane, 15 g of extract was obtained and run into a silica column. The extract was further separated into 220 fractions by silica gel chromatography.

**Figure 4:**
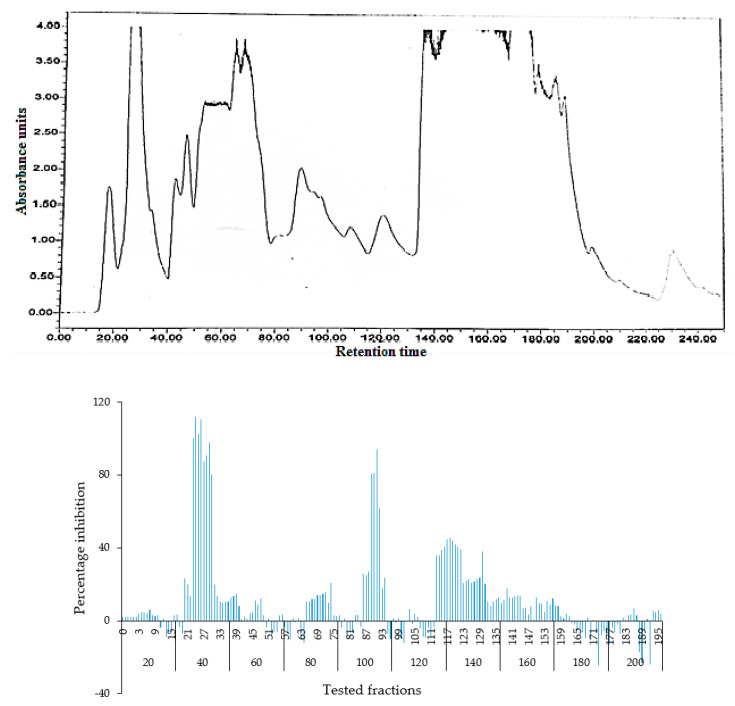
**Top panel**: Overlaid chromatogram of from a silica gel column of the hexane extract of *Bidens bipinnata* leaves; fractions were collected per minute and tested for activity (percentage inhibition of *C. albicans*) (**bottom panel**).

**Figure 5:**
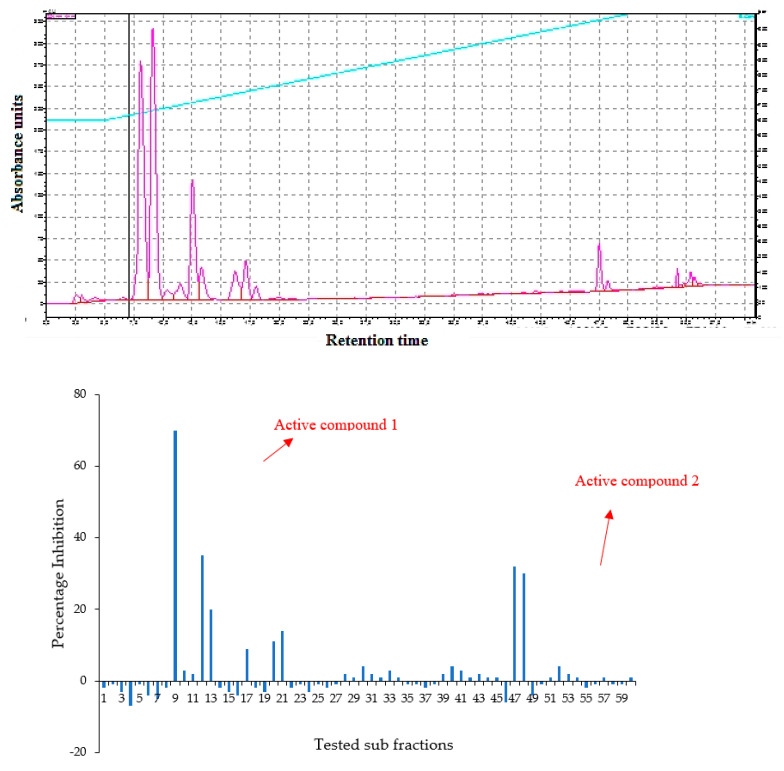
**Top panel**: HPLC chromatogram of fraction 49 of silica gel column; fractions were collected per minute and tested for activity (percentage inhibition of *C. albicans*) (**bottom panel**).

The fractions obtained from the silica column were tested against *C. albicans* and activity was observed in fractions 25–29 and 89–92. All active fractions were dried, weighted and again tested for anticandida activity using a serial dilution protocol and persistent activity was observed in fraction 25–29, indicating the presence of active anticandida compounds (Figure 4). Fraction 27 was further separated using HPLC-DAD Analysis with water: acetonitrile mobile phase (starting at 15% acetonitrile for 5 min and linearly increasing to 100% over 35 min). The chromatogram recorded well-resolved peaks at 254 nm (Figure 5). All 60 one-minute fractions were collected and re-tested for anticandida activity.

**Figure 6:**
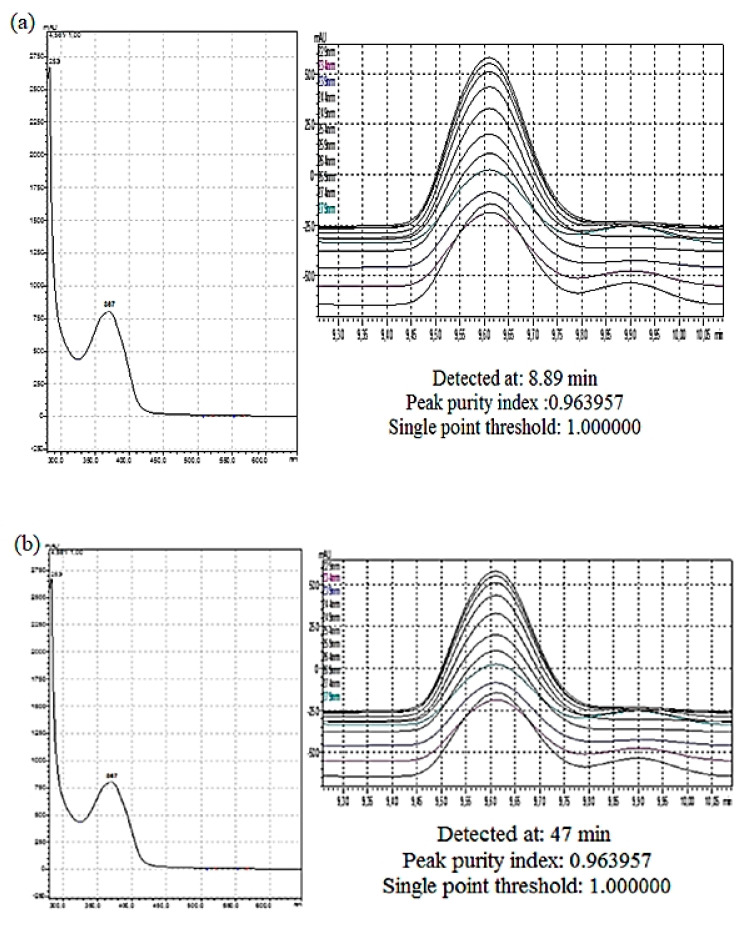
UV-vis apex absorption spectra of (**a**) Compound **1** (Dehydroabietic acid); and (**b**) Compound **2** (Linoleic acid).

**Figure 7:**
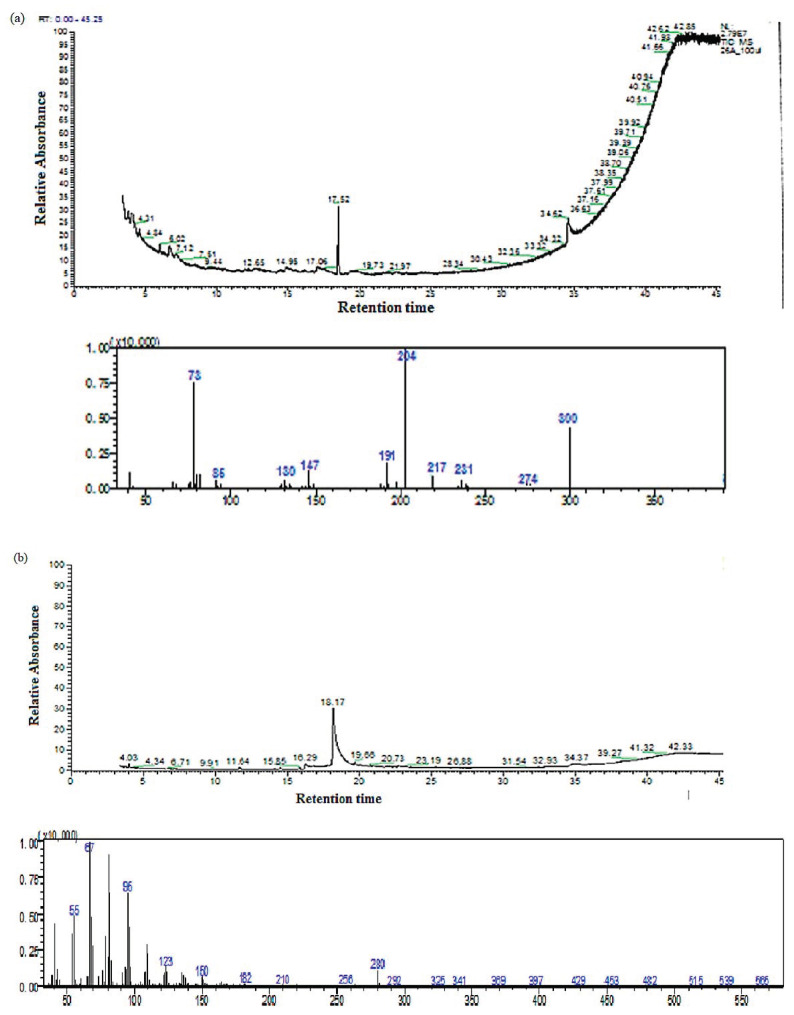
Mass spectra of (**a**) Compound **1** (Dehydroabietic acid) and (**b**) Compound **2** (Linoleic acid).

**Figure 8:**
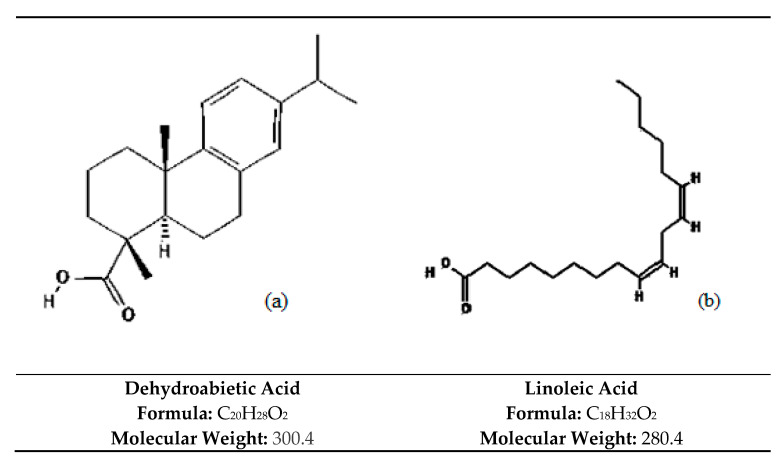
(**a**) Compound **1** (Dehydroabietic acid) and (**b**) Compound **2** (Linoleic acid). Compounds **1** and **2** in the most active peaks were analysed by mass spectrometry.

The purity of the active peaks was tested using TLC and purified active peaks were subjected to UV Vis Spectrophotometry and GCMS for identification (Figure 6 and Figure 7). Compound **1** was identified as dehydroabietic acid with a purity and yield of 94.52 and 54.93%, respectively; compound **2** was identified as linoleic acid with a purity and yield of 92.12 and 65.81%, respectively, (Figure 8). The anticandida activity of both compounds was quantified using a microdilution test on *C. albicans*: linoleic acid had a stronger activity with inhibition of 92% compared to 85% for dehydroabietic acid.

## 3. Discussion

Traditional Chinese Medicine is one of the most common medicinal systems, and various formulations documented in its literature led to the development of modern drugs. In this study, Gui Zhen Cao was evaluated for its pharmacological potential. All three of its plant sources (*B. biternata*, *B. pilosa*, and *B. bipinnata*) were analyzed. These plants showed the presence of phytochemicals, especially tannins, phenols, and alkaloids. Borges et al. [36] indicated that there is a strong correlation between their phenolic content and antioxidant potential. In addition, tannins and alkaloids are thought to be responsible for antimicrobial activity.

During in vitro evaluation it was observed that the hexane extract of *B. bipinnata* appeared to have a notable inhibitory effect against all candida species. Thus, high anticandida activity might be due to the high level of phenols and saponins [36]. In *Handbook of Chinese Medicinal Herbs*, a decoction of Gui Zhen Cao is said to be effective against diarrhea, and since candida species are often considered to be a cause of diarrhea, our results validate its traditional use.

Using bioassay guided fractionation, this study led to the isolation of two anticandida compounds, the most active of which, linoleic acid, was isolated from *B. bipinnata*. The mass spectrum of this compound showed a molecular ion peak at *m*/*z* 280.4 corresponding to the molecular formula C_18_H_32_O_2_. Linoleic acid is a colorless or white polyunsaturated omega-6 fatty acid that is soluble in many organic solvents but nearly insoluble in water. Fatty acids are very important for disease prevention, and linoleic acid can prevent a variety of inflammatory, depressive, cardiovascular, and certain disturbed neurological diseases [37]. It is also reported to decrease serum cholesterol and increase membrane fluidity [38].

The compound had been previously identified from Asteraceae members i.e., *Achillea biebersteinii* Afan [39], *Centaurea vlachorum* [40] and *Achillea gypsicola* [41]. From the genus *Bidens* (*Bidens pilosa* [42] and *B. odorata*)*,* linoleic acid was previously isolated. From *B. bipinnata* there is only one report of its ameliorative effects [43].

In our study, a high anticandida activity of linoleic acid isolated from *B. bipinnata* was reported. Previously linoleic acid was widely reported to have antimicrobial properties. It inhibited the growth of Gram-positive bacterial species with MIC varying between 0.01 and 1.0 mg/mL [37]. In the case of fungal strains it successfully inhibited the growth of the pathogenic fungi *Rhizoctonia solani, Pythium ultimum, Pyrenophora avenae* and *Crinipellis perniciosa* [44]. Greenway and Dyke [45] reported the inhibitory effect of linoleic acid on the growth of *Staphylococcus aureus*, probably by increasing the permeability of the bacterial membrane as a result of its surfactant action. Another study conducted on the effect of linolenic acid against *Helicobacter pylori* suggest that linoleic acid caused structural changes in the microbial cell membrane, thereby distressing its integrity and causing the seepage of cytoplasmic contents [46].

The second-most active compound isolated was dehydroabietic acid with a molecular ion peak at *m*/*z* 300, corresponding to molecular formula C_20_H_28_O_2_. It is a pyran-2,4-dione substituted at position 3 by an acetyl group and at position 6 by a methyl group. It is classified as a pyrone derivative. Pyrones are class of heterocyclic compounds that contain an unsaturated six-membered ring containing a ketone functional group and one oxygen atom. A variety of pyrones were previously isolated from plants: *Gentiana pedicellata* [47], *Hyptis pectinate* [48], *Piper methysticum* [49], *Ravensara anisate* [50] and *Alpinia zerumbet* [51].

Dehydroabietic acid was previously isolated from a variety of plant species: *Abies balsamea* (L.) Mill [52], *Pinus elliottii* [53], *Pinus densiflora* [54], *Nicotiana tabacum* and *Catharanthus roseus* [55]. From Asteraceae members, it was identified from *Commiphora opobalsamum* [56], *Solidago altissima* [57], and *Egletes viscosa* [58]. However, there was no report of this compound from genus *Bidens*.

Dehydroabietic acid is a chief representative of aromatic abietanes. Similar to diterpenoids, these abietans are typically recognized as chemical defense agents. Antiviral, antileishmanial, antifungal, cytotoxic, antitumor, antiulcer, anti-plasmodial, cardiovascular, antimicrobial, antioxidant and anti-inflammatory properties are the biological activities of this group described up to now [59]. Numerous studied have reported the antimicrobial activity of dehydroabietic acid. Franich et al. [60] noticed antifungal activity of its derivatives against *Dothistroma pini.* Feio et al. [61] confirmed that dehydroabietic acid restricted the growth of *Trametes versicolor* (EC50 = 0.04).

In this study, dehydroabietic acid also revealed substantial anticandida activity: 85%. Previous studies indicated its antimicrobial effect [52,54,62] and suggested that dehydroabietic acid may cause a reduction in cell size disrupt cell membranes and cell walls. They also indicated that it decreased the proton gradient in microbial cells [55,56]. This occurrence is related with the disturbance of proton transport in the membrane-bound ATPase, causing uncoupling of the oxidative phosphorylation [63].

## 4. Materials and Methods

### 4.1. Standard and Reagents

Solvents (Ethyl acetate, hexane, acetone, methanol, acetonitrile) of High-Performance Liquid Chromatography(HPLC)-grade purity from Sigma-Aldrich Co. (St. Louis, MO 63118, United States ) deionized sterile water (Milli-Q Reagent Water System, St. Louis, MO, USA), YPD (yeast extract peptone dextrose) agar medium (Lab M Ltd, Lancashire, UK) and DMSO from Sigma-Aldrich (St. Louis, MO, United States) were used.

### 4.2. Collection and Identification of Plants

**Figure 9:**
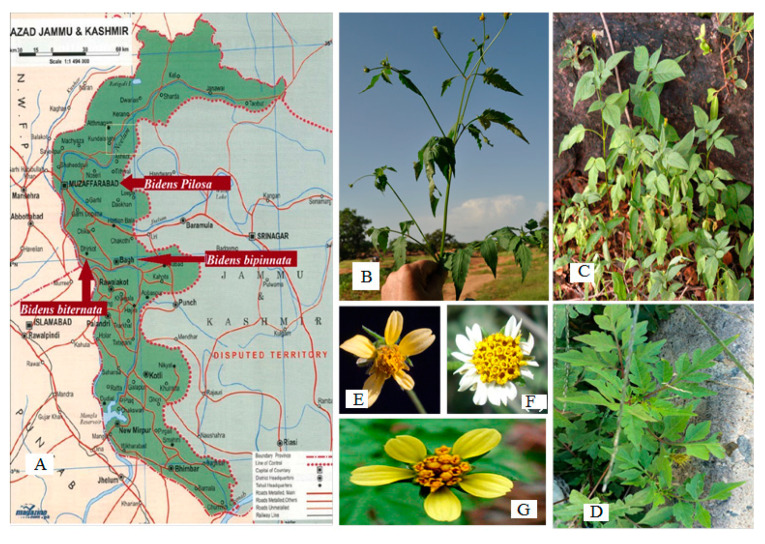
(**A**): Collection areas of Plants; (**B**): *Bidens biternata*; (**C**): *Bidens Pilosa*; (**D**): *Bidens bipinnata*; (**E**): *Bidens biternata* flower; (**F**): *Bidens pilosa* flower; (**G**): *Bidens bipinnata* flower.

*B. pilosa*, *B. biternata*, *B. bipinnata*, was collected from Azad, Jammu and Kashmir, Pakistan, during spring 2017 to spring 2018 (Figure 9). All parts of these plants (root, stem, leaves, flowers and achene’s) were collected in sterile polyethylene bags labeled with the location, name and date of collection. Plant specimens were identified by a taxonomist, and voucher specimens were deposited in the herbarium of Quaid-i-Azam University, Islamabad, Pakistan, for future reference. All parts were thoroughly washed, and diseased or unwanted plant parts were removed. All plant parts were dried at ambient temperature and shade dried to maintain their volatile oils, if present. The plants were fully desiccated to prevent microbial growth and ground into a fine powder, which was kept in in Pearl Pet plastic jars.

### 4.3. Quantitative Analysis of Phytochemicals

#### 4.3.1. Phenolics

By using the Folin-Ciocalteu colorimetric method, total phenolics were determined [64]. Plant extracts were dissolved in methanol, and a 1000 μg/mL solution was prepared. As a standard, Gallic acid was used. Each test solution (300 μL) was taken in a vial and after five minutes, 2.25 mL of Folin-Ciocalteu phenol reagent from Sigma-Aldrich (St. Louis, MO 63118, United States) was added. This mixture was placed in ambient temperature for 90 min and absorbance was taken afterword. Total phenolics were presented as mg Gallic acid equivalent (GAE) per gram.

#### 4.3.2. Tannins

The plant powder was mixed with distilled water (1:2), shaken and filtered. A reagent was prepared by mixing ferric chloride (0.1 N) and ferro-cyanide (0.008 mol) and added to the filtrate, after which absorbance was measured [64].

#### 4.3.3. Alkaloids

Alkaloids were measured using the protocol given by Okumu et al. [65]. The plant powder was mixed with acetic acid, mixed filtered and reduced. Ammonium hydroxide was added, and the precipitates, when formed, were collected, washed, and dried. After that, their weight was measured.

#### 4.3.4. Terpenoids

The plant powder was put into a beaker with 9 mL of ethanol and allowed to stand for 24 h, after which it was filtered, and the filtrate was placed in a separating funnel and 10 mL of petroleum ether was added. The ether extract was then placed in a glass vial and allowed to dry [66]. Once the ether evaporated, the terpenoids were estimated by the following formula:

% Terpenoids = Weight of Tannins/Weight of Extract × 100(1)

#### 4.3.5. Saponins

Using the protocol reported by Ejikeme et al. [64], the plant powder (5 g) was mixed with 20% aqueous ethanol (100 cm^3^) and heated at 55 °C for 4 h in a conical flask in a water bath. The residue was again treated as in the previous step. In a separate funnel, the extract was again treated with 20 cm^3^ of diethyl ether and shaken. Once layer formation occurred, the ether layer was discarded, and the aqueous layer was collected. The procedure was repeated with of *n*-butanol (60 cm^3^) and 5% sodium chloride (10 cm^3^). The *n*-butanol layer was collected and heated in a water bath until dried. The Saponins were calculated as;

% Saponins = Weight of Saponins/Weight of Extract × 100(2)

#### 4.3.6. Oxalates

Oxalates were determined by using the method proposed by Ejikeme et al. [64]. A 2.50 g plant sample was extracted with 0.3 M HCl (20 cm^3^) three times at 50 °C and constantly stirred for 1 h. The extract was treated with 5 M ammonium hydroxide (1.0 cm^3^), 3 drops glacial acetic acid, 2 drops phenolphthalein, and 5% calcium chloride (1.0 cm^3^). The solution was allowed to stand for 3 h and was centrifuged for 15 min at 3000 rpm. Precipitates were collected and washed with hot water three times. In the tube, 3 M tetraoxosulphate acid (2.0 cm^3^) was added, and precipitates were dissolved by warming in a 70 °C water bath. The solution was titrated by 0.01 M potassium permanganate until it turned pink. The solution was left to stand until it again became colorless, after which it was warmed at 70 °C for 3 min and again titrated until the pink colour reappeared and remained for at least 30 s. The oxalates were calculated as;

C_2_O^2−^_4_ + 8H^+^ + MnO^2−^_4_ = 2CO_2_ +4H_2_O + Mn^2+^ (3)

Ratio of reacting ions = 1:1; From M_1_ V_1_ = M_2_ V_2_; M_1_was the molarity of the KMnO_4_; V_1_ was the volume of KMnO_4_; M_2_ was the molarity of the extract; V_2_ was volume of the extract; Molecular weight of CaCO_3_ = 100; Weight of oxalate in titre = *M*_2_ × molecular weight = *Xg*; Weight of oxalate in titrant 2 cm^3^ = (X/100) × 2 = Y; 100 cm^3^ of oxalate extract = (Y/2.5) × 100 g = W

% Oxalate composition g/100 g = (W/2.5) × (100/1)(4)

#### 4.3.7. Cyanogenic Glycoside

In dry wood powder (1 g), distilled water (200 cm^3^) was added and allowed to stand for 2 h. In a conical flask, full distillation was done with 20  cm^3^ of 2.5% NaOH, and tannic acid was used as an antifoaming agent. Ammonium hydroxide 6  M (8  cm^3^) tested extract (100  cm^3^) and 5% potassium iodide was added. The mixture was then titrated with AgNO_3_ (0.02  M). The end point was considered when turbidity occurred [64]. The cyanogenic glycoside was calculated as;

Cyanogenic glycoside (mg/100 g) = (Titrate value (cm^3^) × 1.08 × exact volume/Aliquot Volume (cm^3^) × sample weight (g)) × 100 (5)

#### 4.3.8. Percentage Lipids

Plant powder (2.50  g) was added to a soxhlet extractor and connected to a condenser. Petroleum ether was added, and lipids were extracted by heating at 50 °C. Petroleum ether was removed, and the lipids were recovered by cooling in a desiccator, after which the flask was reweighted [64]. The lipid contents were calculated as;

% Lipid = (Weight of Lipid/Weight of Sample) × 100(6)

### 4.4. In Vitro Anticandida Activity

#### 4.4.1. Fungal Strains

*C. albicans*, *C. glabrata*, *C. parapsilosis*, *C. krusei*, *C. tropicalis*, and *C. orthopsilosis* were used.

#### 4.4.2. Inoculation

A YPD Agar medium (Sigma-Aldrich (St. Louis, MO, United States) as prepared, and colonies of these strains were inoculated and allowed to grow over night at 37 °C. After that, they were stored at 4 °C.

#### 4.4.3. Pre-Culture

The YPD medium was prepared, placed in separate tubes, inoculated with a single colony of each fungal strain and incubated at 37 °C in a shaker incubator Sigma-Aldrich (St. Louis, MO, United States).

#### 4.4.4. Microdilution Broth Protocol

In a 96 well-plate 10 μL of tested plant extract were taken. Ciprofloxacin was taken as a positive control and DMSO and water were taken as a blank control. Each well was then inoculated with 190 μL inoculum (OD = 0.003 at 620 nm). The plates were incubated at 37 °C for 18 h and read on a Mithras LB 940 Multimode Microplate Reader Sigma-Aldrich (St. Louis, MO, United States) at 620 nm.

#### 4.4.5. Small-Scale Extraction

The fully desiccated raw plant material was ground into a fine powder and small-scale extractions were performed as described by Panda et al. [67]. One gram of plant powder was placed into four 15 mL sterile Falcon tubes each with a different solvent: acetone, hexane, ethanol, and water. The tubes were placed in a sonicator bath for 1 h after every 4 h interval. From each extract, 1 mL aliquots were dried in a Savant Speed Vac Concentrator 200 H. The dried filtrate was re-dissolved in 200 mL DMSO for the organic solvent extracts, and in 200 mL water for the aqueous extract. All samples were stored at 4 °C until further testing [67].

#### 4.4.6. Large-Scale Extraction

For large scale extraction, the dried raw botanical material (200 g of powder) was transferred into a large container with 2000 mL of HPLC-grade *n*-hexane from Sigma–Aldrich (Hamburg, Germany). The container was left standing for 24 h at ambient temperature. During this time, the container was placed in a water bath sonicator 4 times for 60 min each to maximize the extraction yield. After each sonication, there was an interval of at least 6 h to let the suspension cool to ambient temperature. After the fourth sonication, the material was filtered using a VWR^®^ Grade 313 with 5 μm filter paper (from Sigma–Aldrich (Hamburg, Germany) to obtain a dry residue. The filtrate was then evaporated in a rotary evaporator (BUCHI rotavapor R-100, Sigma-Aldrich (St. Louis, MO 63118, United States) and the weight of the first dried extract was measured. The recovered hexane was again used for the second extraction using same procedure for 24 h, after which the filtrate was again evaporated with a rotary evaporator to obtain dried extract. The same procedure was repeated to derive all the compounds from the plant powder. The final weight of the dried material was calculated and the plant extract was stored at 4 °C for further analysis.

The plant powder was dried again so it could be treated with a polar solvent to derive a polar compound with the same procedure.

#### 4.4.7. Fractionation Procedure

The dried active extract was adsorbed onto silica gel (70–230 mesh) and loaded on a silica gel column (600 mm height × 55 mm diameter) Sigma–Aldrich (Hamburg, Germany) which was eluted (Waters Gradient Chromatography Calculator, model 600, Sigma–Aldrich (Hamburg, Germany) with a step gradient of hexane–dichloromethane that increased in polarity: 9.5:0.5, 9:1, 8.5:0.5, 8:2, 7:3; 6:4, 5:5, 4:6, 3:7, 2:8, 1:9, and 0:10. The column was then eluted with 100% dichloromethane, and then 100% ethyl acetate, followed by a mixture of ethyl acetate and methanol (9.5:0.5, 9:1, 8:2, 7:3, 6:4, 5:5, 4:6, 3:7, 2:8, and 1:9). Finally, the column was eluted with 100% methanol. In each step, 10 tubes of 40 mL fractions were collected, and the solvent evaporated. The whole separation was monitored by a Dual λ model 2487 absorbance detector (Waters Milford, MA, USA) at 280 nm and 254 nm.

#### 4.4.8. Thin Layer Chromatography

Aliquots (5 µL) of the 245 fractions from the ethyl acetate extract were spotted on large TLC glass plates (Sigma-Aldrich, Hamburg Germany, dimensions 20 cm × 20 cm). The spotted plates were developed in glass jars (20 cm × 10 cm × 20 cm), pre-saturated with the chosen mobile phase at ambient temperature and dried in an oven (St. Louis, MO 63118, United States) at 90 °C for 5 min to remove the solvent. The plates were observed under ultra-violet (UV) light (Sigma-Aldrich, Hamburg Germany) at 254 and 360 nm, followed by spraying with 5% sulfuric acid in ethanol, followed by heating at 100 °C for 5 min. Fractions with similar TLC patterns were pooled for biological activity testing. In total, 22 pooled fractions were prepared and further tested for antimicrobial activity against *C. albicans* at a concentration of 1000 µg/mL.

#### 4.4.9. HPLC-DAD Analysis

HPLC analyses were performed on a Shimadzu, LC-20AT system (model DGU 20A3, SHIMADZU corporation, Kyoto, Japan) equipped with LC-20AT quaternary pump, a DGU20A3/DGU-20A5 on-line degasser, an SPD-20A photodiode array detector, and a CBM-20A/20A interface. The data were acquired and processed using Lab Solution software. The active fraction was analyzed using a reverse-phase HPLC column SHIMADZU corporation, Kyoto, Japan), SunfireTM prep C18 (10 mm × 250 mm, 5 µm) column (Waters, Ireland). The mobile phase was composed of 30% H_2_O with 0.1% TFA (ACROS ORGANICS) and 70% acetonitrile (LC-MS CHROMASOLVR, Fluka) with 0.1% TFA. Prior to HPLC injection, samples were filtered through a CHROMAFIL R Xtra H-PTFI filter (pore size 0.45 µm, filter 13 mm, (MACHEREY-NAGEL, Düren, Germany). Two mL of sample was injected and run for 41 min at 20 °C with a flow rate of 4 mL/min [68].

#### 4.4.10. Mass Spectrometry GC-MS

Collected peaks were analysed on a gas chromatograph (Thermo–Fisher Scientific Trace 1300 series, (Thermo Finnigan LLC, San Jose, CA, United States) coupled with a mass spectrometer (Thermo–Fisher Scientific ISQ series MS, Thermo Finnigan LLC, San Jose, CA, United States, The column used was a Restek RXi-5sil MS 20 m with an internal diameter of 0.18 mm and a film thickness of 0.18 µm. Helium was used as a carrier gas at a constant flow rate of 0.9 mL/min. The initial temperature of 40 °C was held for 2 min, then increased to 120 °C at a rate of 20 °C/min, to 200 °C at a rate of 10 °C/min, to 250 °C at a rate of 7 °C/min, and finally to 350 °C at a rate of 5 °C/min, which was held for 4 min. The spectrum corresponding to the largest peak in the chromatogram was searched against the NIST 14 MS library for identification. In addition, the identified compound was verified for its retention index, which was calculated using an external C7 to C40 alkane ladder.

## 5. Conclusions

In conclusion, the hexane extracts of *B. bipinnata* leaves showed anticandida activity due to the presence of linoleic acid and dehydroabietic acid. This supports the traditional use of Gui Zhen Cao. It is suggested that isolated compounds should be studied against different pure culture cell lines to identify their potential in vivo toxicity and also to understand their mode of action. In addition the activity of linoleic and dehydroabietic acids, with different combinations of compounds, must be studied to suggest possible synergistic effects.

## Figures and Tables

**Table 1:** Evidence of Gui Zhen Cao medicinal potential In Chinese Traditional medicine.

Book Name	Targets of Gui Zhen Cao Curative Properties	Reference
*Oriental Meteria Medica*	Wind-dampness, dispersing stagnant blood, and invigorating blood.	[3]
*Handbook of Chinese Medicinal Herbs*	Dysentery, laryngalgia, dysphagia, vomiting, cardiac spasm and esophageal dilatation.	[4]
*Prescriptions Worth A Thousand Gold*	Blended with pig fat to cure finger cuts.	-
*Chinese-English Manual of Common-Used Herbs*	Common cold of the wind-heat type, influenza; clear away heat and toxic materials: sore throat, appendicitis, snake bite, and centipede bite.	[5]
*Thousand Formulas and Thousand Herbs of Traditional Chinese Medicine*	Heat from gastro-intestinal tract: for diarrhea, dysentery and stomach ache of heat type.	[6]

**Table 2:** Ethnomedicinal uses of Gui Zhen Cao herbs.

Disease	Plant Used	Mode of Application	Reference
Stomach ache	*B. pilosa*	Decoction of fresh leaves	[14]
	*B. bipinnata*	Not stated	[15]
Diarrhea	*B. pilosa*	Decoction and fresh leaves	[16]
	*B. bipinnata*	Not stated	[17]
Anti-inflammatory	*B. pilosa*	Not stated	[18]
	*B. biternata*	Poultice of leaf	[19]
Dysentery	*B. pilosa*	Decoction of whole plant	[20]
	*B. biternata*	Not stated	[21]
	*B. bipinnata*	Not stated	[17]
Headache	*B. pilosa*	Decoction of whole plant	[22]
	*B. biternata*	Bruised leaves on forehead	[23]
Colds	*B. pilosa*	Fresh leaves or decoction of whole plant	[24]
	*B. biternata*	Decoction of whole plant	[25]
Eye Infection	*B. pilosa*	Juice of fresh leaves used as eye and ear drops	[26]
	*B. biternata*	Same as above	[27]
Wounds	*B. pilosa*	Crushed herb	[28]
	*B. biternata*	Leaves rubbed as a hemostatic	[29]
Snake bite	*B. pilosa*	Pulverized herb	[30]
	*B. biternata*	Fresh roots paste is given as a drink	[19]
Toothache	*B. biternata*	Roots are chewed	[21]
Cough	*B. pilosa*	Decoction of whole plant is taken orally	[31]
	*B. biternata*	Infusion is given	[27]
Stomach ulcers	*B. pilosa*	Maceration or juice; taken orally	[32]
Tuberculosis	*B. biternata*	Decoction or maceration; taken orally	[29]
Vaginitis	*B. pilosa*	Decoction of fresh leaves are applied	[12]
	*B. bipinnata*, *B. pilosa*	Not stated	[33]
Candidiasis	*Bidens biternata*	Essential oil	[34]
Skin infections	*B. pilosa*	Ground leaves	[35]
	*B. bipinnata*	Not stated	[17]

**Table 3:** Quantity of Phytochemicals found in *Bidens* species.

Plant Sample		Tannin (mg/100 g)	Alkaloid (%)	Flavonoid (%)	Saponins (%)	Oxalate (%)	Cyanogenic Glycoside (mg/100 g)	Phenols (mg/g)	Lipid (%)
*Bidens biternata*	Vegetative parts	1090 ± 1.4142	0.499	6.9	3.6	2.21	620 ± 0.836	3.14 ± 0.5468	8.6
	Reproductive parts	956 ± 2.8280	0.234	9.2	6.6	2.08	501 ± 1.927	4.09 ± 0.5463	2.8
*Bidens bipinnata*	Vegetative parts	930 ± 4.7140	0.231	5.09	7.09	2.97	520 ± 0.275	4.04 ± 1.6750	6.4
	Reproductive parts	865 ± 0.9428	0.201	8.2	9.8	1.76	465 ± 1.3568	5.98 ± 0.7979	5.76
*Bidens pilosa*	Vegetative parts	1030 ± 0.9436	0.357	6.98	8.32	3.05	500 ± 0.6571	3.58 ± 0.1454	7.2
	Reproductive parts	827 ± 0.9428	0.214	7.6	9.8	2.09	487 ± 0.2468	4.98 ± 0.7564	6.54

## Data Availability

The data presented in this study are available upon fair request from the corresponding author.

## Sample Availability

Not available.
